# Coordinated Acetylcholine Release in Prefrontal Cortex and Hippocampus Is Associated with Arousal and Reward on Distinct Timescales

**DOI:** 10.1016/j.celrep.2016.12.085

**Published:** 2017-01-24

**Authors:** Leonor M. Teles-Grilo Ruivo, Keeley L. Baker, Michael W. Conway, Peter J. Kinsley, Gary Gilmour, Keith G. Phillips, John T.R. Isaac, John P. Lowry, Jack R. Mellor

**Affiliations:** 1 Lilly Centre for Cognitive Neuroscience, Eli Lilly and Company Ltd., Erl Wood Manor, Windlesham, Surrey GU20 6PH, UK; 2 Centre for Synaptic Plasticity, School of Physiology, Pharmacology and Neuroscience, University of Bristol, Bristol BS8 1TD, UK; 3 Department of Chemistry, Maynooth University, Co. Kildare, Ireland

**Keywords:** acetylcholine, hippocampus, prefrontal cortex, biosensor

## Abstract

Cholinergic neurotransmission throughout the neocortex and hippocampus regulates arousal, learning, and attention. However, owing to the poorly characterized timing and location of acetylcholine release, its detailed behavioral functions remain unclear. Using electrochemical biosensors chronically implanted in mice, we made continuous measurements of the spatiotemporal dynamics of acetylcholine release across multiple behavioral states. We found that tonic levels of acetylcholine release were coordinated between the prefrontal cortex and hippocampus and maximal during training on a rewarded working memory task. Tonic release also increased during REM sleep but was contingent on subsequent wakefulness. In contrast, coordinated phasic acetylcholine release occurred only during the memory task and was strongly localized to reward delivery areas without being contingent on trial outcome. These results show that coordinated acetylcholine release between the prefrontal cortex and hippocampus is associated with reward and arousal on distinct timescales, providing dual mechanisms to support learned behavior acquisition during cognitive task performance.

## Highlights

• Acetylcholine release is coordinated in prefrontal cortex and hippocampus
• Tonic and phasic release are maximal during training on a cognitive task
• Tonic acetylcholine release during REM sleep predicts subsequent wakefulness
• Phasic acetylcholine release is preferentially associated with reward

## Graphical Abstract

**Figure:**
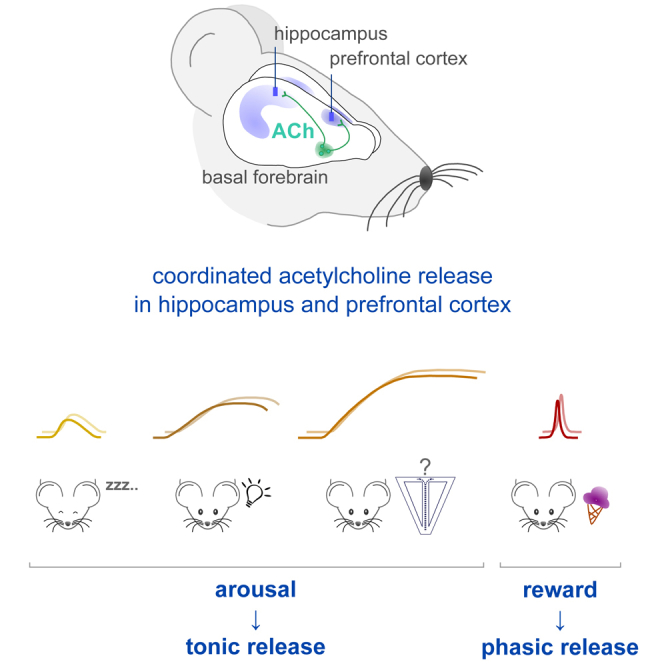

## Teaser

In this study, Teles-Grilo Ruivo et al. use biosensors to simultaneously measure the release profiles of the neuromodulator acetylcholine in the prefrontal cortex and hippocampus of mice. They find that release on both tonic and phasic timescales is remarkably coordinated between brain regions and dependent on behavioral state.

## Introduction

Cholinergic neurons in the basal forebrain (BF) and medial septum/diagonal band of Broca (MS-DBB) innervate cortical and subcortical structures, including the prefrontal cortex and hippocampus, respectively (Mesulam et al., 1983). These projections play an important role in attention and memory processes (Hasselmo and Sarter, 2011), likely by desynchronizing neuronal networks to enhance the signal-to-noise ratio for salient information (Chen et al., 2015, Everitt and Robbins, 1997, Fu et al., 2014, Harris and Thiele, 2011, Hasselmo, 2006, Pinto et al., 2013). De-innervation of cholinergic afferents results in attentional deficits and reduced vigilance (McGaughy et al., 2000), and stimulation of cholinergic afferents can also produce reinforcement of behavior triggered by rewarding or aversive stimuli (Hangya et al., 2015, Liu et al., 2015). Acetylcholine release is also critical for switching neuronal networks into high-arousal states that are similarly characterized by less synchronized activity (Saper et al., 2010). However, the precise timing and location of acetylcholine release have remained unclear, leaving open the question of whether cholinergic nuclei function in a coordinated or an independent manner and by what mechanisms and timescales acetylcholine release regulates arousal, attention, or reinforcement learning (Teles-Grilo Ruivo and Mellor, 2013).

Microdialysis studies have shown acetylcholine release in neocortex and hippocampus increases during attention, stress, exploration, and locomotion (Pepeu and Giovannini, 2004) and that acetylcholine levels are high during REM sleep but low during slow-wave or non-REM (NREM) sleep (Marrosu et al., 1995). However, the limited temporal resolution of microdialysis prevents detection on a sub-minute timescale that is most relevant to many cognitive processes and, furthermore, leaves open the question of whether fluctuations in acetylcholine are mediated by an increase in non-synchronized release from multiple presynaptic boutons over a period of minutes (tonic release) or highly synchronized release within a few seconds (phasic release) (Sarter et al., 2009).

Higher temporal-resolution measurements of cholinergic neuron activity by juxtacellular recording or calcium imaging show low basal firing rates (Lee et al., 2005, Simon et al., 2006) that increase in MS-DBB neurons projecting to the hippocampus during aversive stimuli (Lovett-Barron et al., 2014) or in BF neurons projecting to the neocortex during whisking (Eggermann et al., 2014, Nelson and Mooney, 2016), waking, and REM sleep (Lee et al., 2005). However, juxtacellular recordings or calcium imaging necessarily restrict movement; the duration of recordings; and, therefore, the range of behavioral states tested. Alternative approaches using optogenetic identification of extracellularly recorded cholinergic neuron activity reveal that cholinergic neurons are activated in response to rewarding or aversive cues, suggesting a role in reinforcement of behavior (Hangya et al., 2015), but this method does not distinguish where acetylcholine is subsequently released. To overcome these limitations and investigate the spatiotemporal dynamics of acetylcholine release across a range of behavioral states and brain regions in freely moving animals with sub-second temporal resolution, we made use of electrochemical enzyme-based biosensors (Baker et al., 2015, Bruno et al., 2006a, Parikh et al., 2004, Parikh et al., 2007, Zhang et al., 2010). This technique enables the measurement of extracellular levels of acetylcholine not confined to the synaptic cleft and, to date, has only been used to investigate phasic acetylcholine release in the medial prefrontal cortex (mPFC), where it was found to be involved in the processes of cue detection (Parikh et al., 2007).

Using constant potential amperometry and electrochemical enzyme-based biosensors selective for choline—and, therefore, an accurate readout of acetylcholine release (Baker et al., 2015, Bruno et al., 2006a, Parikh et al., 2004, Parikh et al., 2007)—tonic and phasic release of acetylcholine were measured simultaneously in the mPFC and dorsal hippocampus (dHPC) of young adult mice. We find that tonic acetylcholine release is coordinated in the mPFC and dHPC and predicts the transition of behavior between different arousal states. In contrast, phasic acetylcholine release is found only during performance on a working memory task, where it is strongly associated with the reward delivery areas in both the mPFC and dHPC. Thus, our data support a role for acetylcholine release in arousal and reward signaling on multiple timescales.

## Results

To measure the spatiotemporal dynamics of acetylcholine release, choline biosensors were co-implanted in the mPFC and dHPC of mice (Figure S1). It has been confirmed by several groups, using local pressure ejections, perfusions of choline/acetylcholine, and compounds known to increase/decrease cortical acetylcholine efflux (e.g., KCl, scopolamine, and neostigmine), that, at a potential of +700 mV, biosensors reliably detect acetylcholine release by measuring choline produced by endogenous acetylcholinesterase (Baker et al., 2015, Bruno et al., 2006a, Parikh et al., 2004, Parikh et al., 2007). In addition, their improved temporal resolution (e.g., sub-second; Bruno et al., 2006b, Burmeister et al., 2008, Lowry et al., 1994, Lowry et al., 1998) and spatial resolution (e.g., <200 μm) over techniques such as microdialysis facilitate studies relating transmission to responses associated with individual stimuli and behavior and can discriminate heterogeneities within brain regions (McHugh et al., 2011, Parikh et al., 2004). Biosensors are also specifically designed to maximize substrate sensitivity and to restrict access to other neurotransmitters and potential endogenous electroactive interferents (see Experimental Procedures).

In vitro characterization studies confirmed minimal interference from endogenous electroactive species (e.g., ascorbic acid, dopamine, serotonin, and their metabolites 3,4-dihydroxyphenylacetic acid and 5-hydroxyindoleacetic acid; K.L.B. and J.P.L., unpublished data). Typical data for ascorbic acid, which is regarded as the principal electroactive interferent (Brown and Lowry, 2003, Garguilo and Michael, 1995), as it has a high basal level (ca. 300–500 μM) and a continuously changing extracellular concentration (O’Neill, 1995), are shown in Figure S2B. Such interference rejection characteristics have also recently been validated in vivo (Baker et al., 2015) and are similar to those previously observed for PPD (polymerized phenylenediamine)-based glucose biosensors (Lowry et al., 1998, Lowry and O’Neill, 1994).

Similar classic biosensor designs have been developed and used successfully by several groups for monitoring a variety of neurochemicals in vivo, including glucose, lactate, and glutamate (Boutelle et al., 1986, Dash et al., 2013, Hu et al., 1994, Hu and Wilson, 1997). The increased surface area used in such designs typically negates the need for the use of a self-referencing sentinel electrode that is typical of microelectrode array biosensor designs that have a planar geometry (e.g., 15 μm × 333 μm [Parikh et al., 2007] or 50 μm × 150 μm [Zhang et al., 2010]) and significantly lower sensitivity (ca. 19 pA/μM; Parikh et al., 2004). The increased sensitivity in the larger sensors used here would most likely result in cross-talk at the sentinel electrode from diffusion of the surface-generated hydrogen peroxide (H_2_O_2_) out from the enzyme layer (Vasylieva et al., 2015). Recent miniaturization of the classic design highlights the importance of the sentinel electrode when sensitivity is reduced (6.4 pA/μM), and electrophysioloical signals from local field potentials (LFPs) are extracted from the high-frequency (>1 Hz) component of the amperometric biosensor signal (Santos et al., 2015).

**Figure 1:**
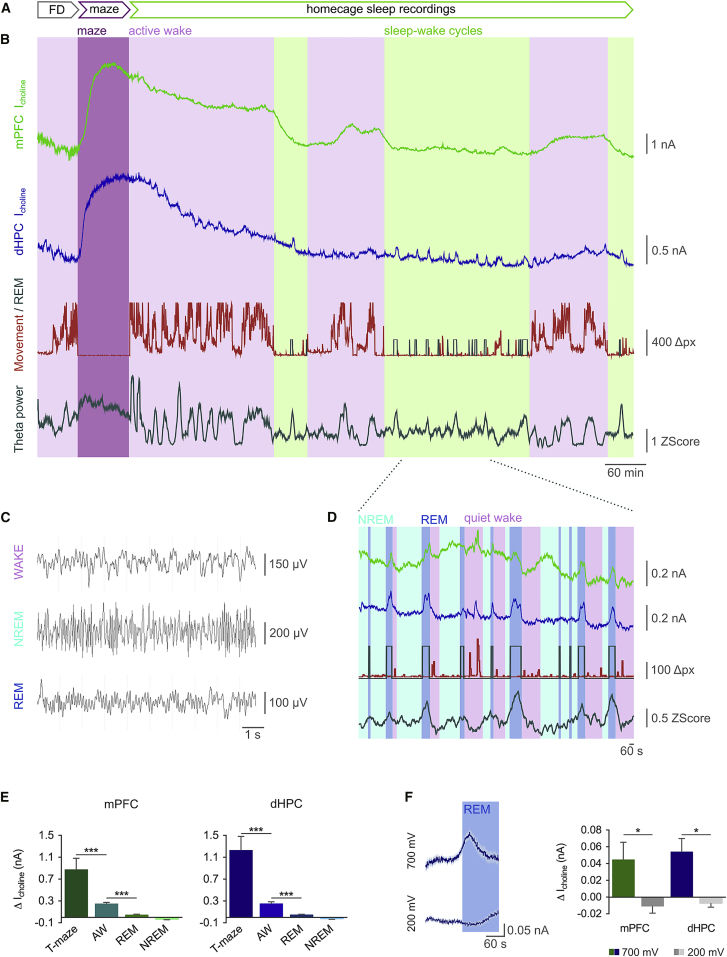
Tonic Acetylcholine Release Is Associated with Arousal (A) Experimental timeline. Biosensor and LFP electrodes were implanted in 6-week-old mice. Following a week of recovery, sleep recordings and training on a randomized forced-alternation T-maze were performed daily over 5 consecutive days. FD, food deprivation (overnight). (B) Continuous recordings of choline current (I_choline_) in mPFC and dHPC with corresponding movement, REM, and z-scored theta power. Background shading highlights example epochs of designated behavioral states. (C) Example LFP traces during wake, NREM sleep, and REM sleep. (D) Detail of acetylcholine release during sleep-wake cycles. (E) Quantification of changes in choline current measured during maze, active wakefulness (AW), REM, and NREM (n = 6 mice; ANOVA with Tukey HSD post hoc correction). (F) Control experiments with biosensor potential at +200 mV show no increase in current during REM (n = 4 mice; paired t test). Data are indicated as mean ± SEM. ^∗^p < 0.05; ^∗∗∗^p < 0.001.

In these experiments, recordings were performed continuously in the homecage and during the first 5 days of training on a randomized forced alternation T-maze working memory task (Figures 1A, 1B, 3A, and 3B) (Kucewicz et al., 2011). Sleep and wake states were determined by simultaneous recording of hippocampal LFP and locomotor activity combined with the automated sleep scoring algorithm based on SCORE (Van Gelder et al., 1991) (Figures 1B and 1C). By this method, states were designated as active or quiet wakefulness and REM or NREM sleep. Epochs classified as sleep often contained multiple REM and NREM episodes interleaved with quiet wakefulness (Figure 1D). Tonic and phasic release are here referred to, respectively, as desynchronized firing of cholinergic terminals on the scale of tens of seconds to minutes, leading to a slow changing, sustained extracellular cholinergic signal; and as synchronous firing across the population of cholinergic inputs, generating fast extracellular acetylcholine transients detected on the scale of milliseconds to seconds. These release profiles were clearly distinguished with halfwidths >30 s (tonic) and <5 s (phasic) (Sarter et al., 2009).

### The Spatiotemporal Dynamics of Tonic Acetylcholine Release across Sleep-Wake Cycles

Microdialysis studies have found that acetylcholine concentrations in cortical and hippocampal brain regions are high during locomotion and performance of navigation- or attention-based tasks (Dalley et al., 2001, Giovannini et al., 2001, Pepeu and Giovannini, 2004), but it is unclear whether acetylcholine concentrations fluctuate on a faster timescale than may be resolved using microdialysis. Using biosensors with a temporal resolution of <1 s (Baker et al., 2015, Bruno et al., 2006b, Burmeister et al., 2008, Lowry et al., 1994, Lowry et al., 1998), we found that tonic acetylcholine concentration increased monotonically on a slow timescale (>5 min) and was maximal in both the mPFC and dHPC during training on a novel behavioral task (Figures 1B and 1E; mPFC, 0.88 ± 0.21 nA; dHPC, 1.23 ± 0.25 nA). The average maximum increase in choline concentration during the task was approximately 1.3 μM in the mPFC and 1.9 μM in the dHPC, calculated from the current-concentration calibration performed in vitro for each biosensor (Baker et al., 2015) (Figure S2C). This was 3- to 4-fold higher in comparison with periods of active wakefulness in the homecage, which included periods of hyperactivity observed before maze training as a result of overnight food deprivation. Increased acetylcholine release during maze training was not solely a result of increased locomotor activity (Giovannini et al., 2001), as there was only a weak correlation observed between locomotor activity and maximum choline current during periods of active wakefulness (Figure S3A) and no correlation between running speed on the maze and acetylcholine release. Locomotor activity was lowest in the holding area and highest in the middle and turning arms; however, the recorded choline current remained stable across time within a single training session (Figure S3B) and across training days (Figures 3D and 3E; Figure S3C).

**Figure 2:**
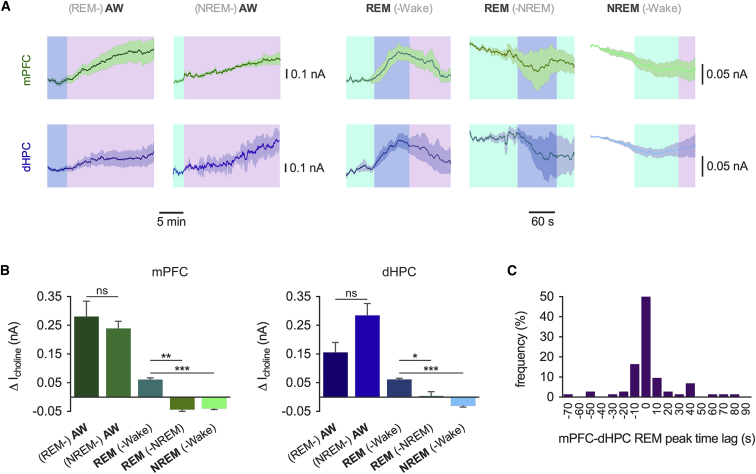
The Dynamics of Tonic Acetylcholine Release Predict Behavioral Sequences (A) Example choline currents for each behavioral state sequence. Background shading is color coded for behavioral state (AW, active wakefulness), measured state is in black, and preceding or following state is in gray. See Table 1. (B) Quantification of choline current changes for behavioral state sequences shown in (A) (n = 6 mice; ANOVA with Tukey HSD post hoc correction). Data are indicated as mean ± SEM. (C) Frequency distribution of the time lag, in seconds, between mPFC and dHPC REM (-Wake) choline peaks from REM onset. ^∗^p < 0.05; ^∗∗^p < 0.01; ^∗∗∗^p < 0.001; ns, p > 0.05.

Previous reports have shown that tonic acetylcholine release measured by microdialysis in cat hippocampi is lowest during NREM sleep, higher during active wakefulness and highest during REM sleep (Marrosu et al., 1995). In contrast, mouse mPFC and dHPC showed small, defined increases in acetylcholine during most REM sleep epochs matching the rise in power of theta frequency oscillations in the hippocampal LFP typical of REM sleep (Figures 1D and 1E). The increase in acetylcholine release during REM sleep was smaller than during active wake. This was also true if only REM epochs followed by wakefulness, and not nested within NREM sleep, were considered (Figure 2). Importantly, control recordings performed at an applied biosensor potential of +200 mV, at which choline currents (from choline-oxidase [ChOx]-generated H_2_O_2_) are not detected (Figure S2D), confirmed that the increases in choline current were a result of acetylcholine release and not interference from other electrochemical species (e.g., ascorbic acid, dopamine, serotonin, and their metabolites), which would typically oxidize at this potential (K.L.B. and J.P.L., unpublished data) (Figure 1F).

### Tonic Acetylcholine Release Predicts Behavioral State Transitions

We next tested whether tonic acetylcholine release was related to the sequence of behavioral states. Typical sleep patterns involve transition from wakefulness into NREM sleep followed by cycles of NREM-REM with transitions back to wakefulness from either sleep state. This means REM sleep can transition to wakefulness or NREM states but is normally always preceded by periods of NREM sleep. Increases in acetylcholine release during active wakefulness were similar regardless of preceding sleep state (Figures 2A and 2B), but, interestingly, although acetylcholine release during NREM sleep was consistently low, acetylcholine only increased during REM sleep if it was followed by a period of wakefulness. Both these observations were consistent across brain structures (Figures 2A and 2B). Indeed, 68.5% of REM events saw a coordinated increase in acetylcholine release in the mPFC and dHPC (signal peaks within 10 s) (Figure 2C) without a preference for the increase in one brain region to precede the other (mPFC, 43.2%; dHPC, 56.9%). Analysis of the proportion of REM epochs where acetylcholine increased for either REM followed by wake (REM-Wake) or NREM (REM-NREM) revealed that almost all REM-Wake epochs had acetylcholine increases, whereas very few REM-NREM epochs did (mPFC, 87.6% ± 7.9% versus 15.3% ± 8.2%, p < 0.01; dHPC, 76.2% ± 15.8% versus 13.9% ± 9.0%, p < 0.05). Therefore, acetylcholine increase during REM sleep is a predictor of subsequent wakefulness.

**Figure 3:**
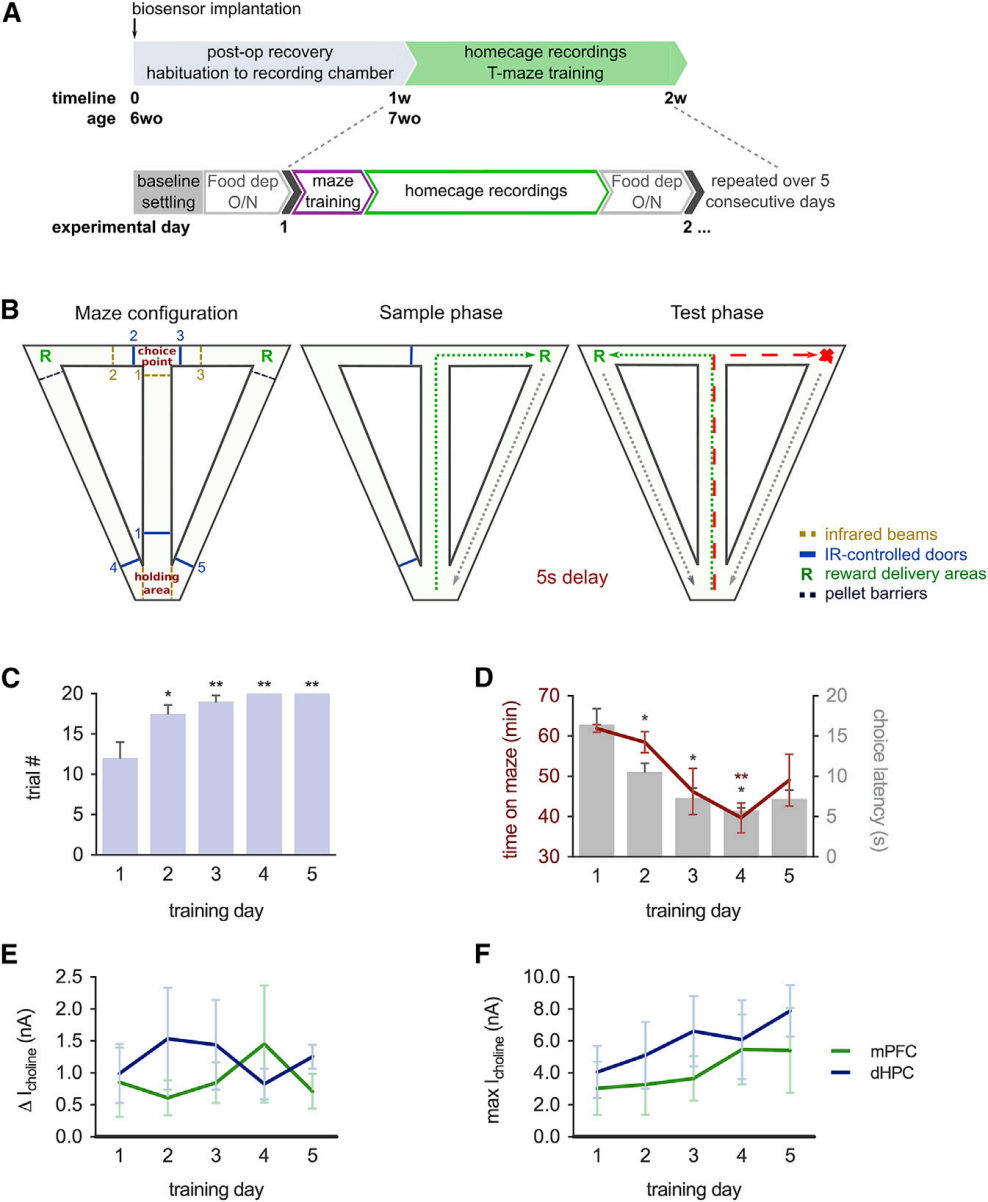
Tonic Acetylcholine Release Is Consistent across Training Days on a Spatial Memory Task (A) Experimental timeline. Sleep recordings and training on the T-maze were performed daily over 5 consecutive days, 1 week after biosensor implantation. op, operation; wo, weeks old; dep, deprivation; O/N, overnight. (B) Automated T-maze configuration. Animals make a forced turn and are given a reward (sample phase) and, after a 5-s delay, must make a choice turn and receive a reward when the alternate arm is chosen (test phase). IR, infrared. (C) The number of trials completed per training session increased over the training period (n = 6 mice). ^∗^p < 0.05 and ^∗∗^p < 0.01, denoting pairwise comparisons with day 1, ANOVA with Tukey HSD post hoc correction. (D) The choice latency and the time taken to complete the maximum number of trials decreased (n = 6 mice). ^∗^p < 0.05 and ^∗∗^p < 0.01, denoting pairwise comparisons with day 1, Welch ANOVA with Games-Howell post hoc correction. (E and F) Tonic acetylcholine release (E) and maximum choline levels (F) associated with maze training were stable over consecutive training days (n = 6 mice). All pairwise comparisons, ns (p > 0.05), ANOVA with Tukey HSD post hoc correction. Data are indicated as mean ± SEM.

Animals were tested on a T-maze spatial working memory task (Figures 3A and 3B) that requires both the HPC and PFC and is supported by the direct connection between them (Ainge et al., 2007, Jones and Wilson, 2005, Kucewicz et al., 2011, Spellman et al., 2015). Performance on the maze improved during training measured by an increase in the number of completed trials in 1 hr and a shortening of the time taken to complete the maximum number of trials and the choice latency time (Figures 3C and 3D). The decrease in total time spent on the maze was primarily due to an increase in the running speed across training days (Figure S3C). The percentage of correct trials remained constant over consecutive training days. The increase in acetylcholine release during maze training was consistent across consecutive training days in both the mPFC and dHPC (Figure 3E). The maximum choline current measured during maze training was also mainly consistent, although there was a small non-significant trend toward increase over the 5-day training period, indicating a small increase in baseline acetylcholine concentration measured immediately prior to maze training (Figure 3F). The consistency of tonic acetylcholine release, and its dissociation from locomotor-activity-dependent changes during maze training, suggests that it is important for efficient maze performance by enhancing vigilance state.

### Phasic Acetylcholine Release during a Spatial Memory Task

Theories of the modality of cholinergic transmission have recently been revised from tonic volume transmission based on the observed low firing rates of cholinergic neurons and anatomically diffuse projections (Lee et al., 2005, Mesulam et al., 1983, Simon et al., 2006, Teles-Grilo Ruivo and Mellor, 2013) to include phasic transmission events that result from the synchronized firing of cholinergic neurons and release of acetylcholine on a timescale of <1 s (Sarter et al., 2009). Phasic transmission has been shown to occur in the neocortex during active whisking (Eggermann et al., 2014, Nelson and Mooney, 2016), in the HPC in response to fear conditioning (Lovett-Barron et al., 2014), and in the PFC as a signal for cue detection (Parikh et al., 2007). The activity of BF cholinergic neurons responds to both reward and aversive stimuli (Hangya et al., 2015), but it is not clear whether phasic acetylcholine release occurs during other cognitive tasks, in the absence of a cognitive challenge, or whether phasic release is coordinated between brain regions in a similar manner to tonic release. Therefore, we next tested whether and when phasic acetylcholine release occurs across sleep-wake cycles and during performance on the T-maze spatial working memory task.

**Figure 4:**
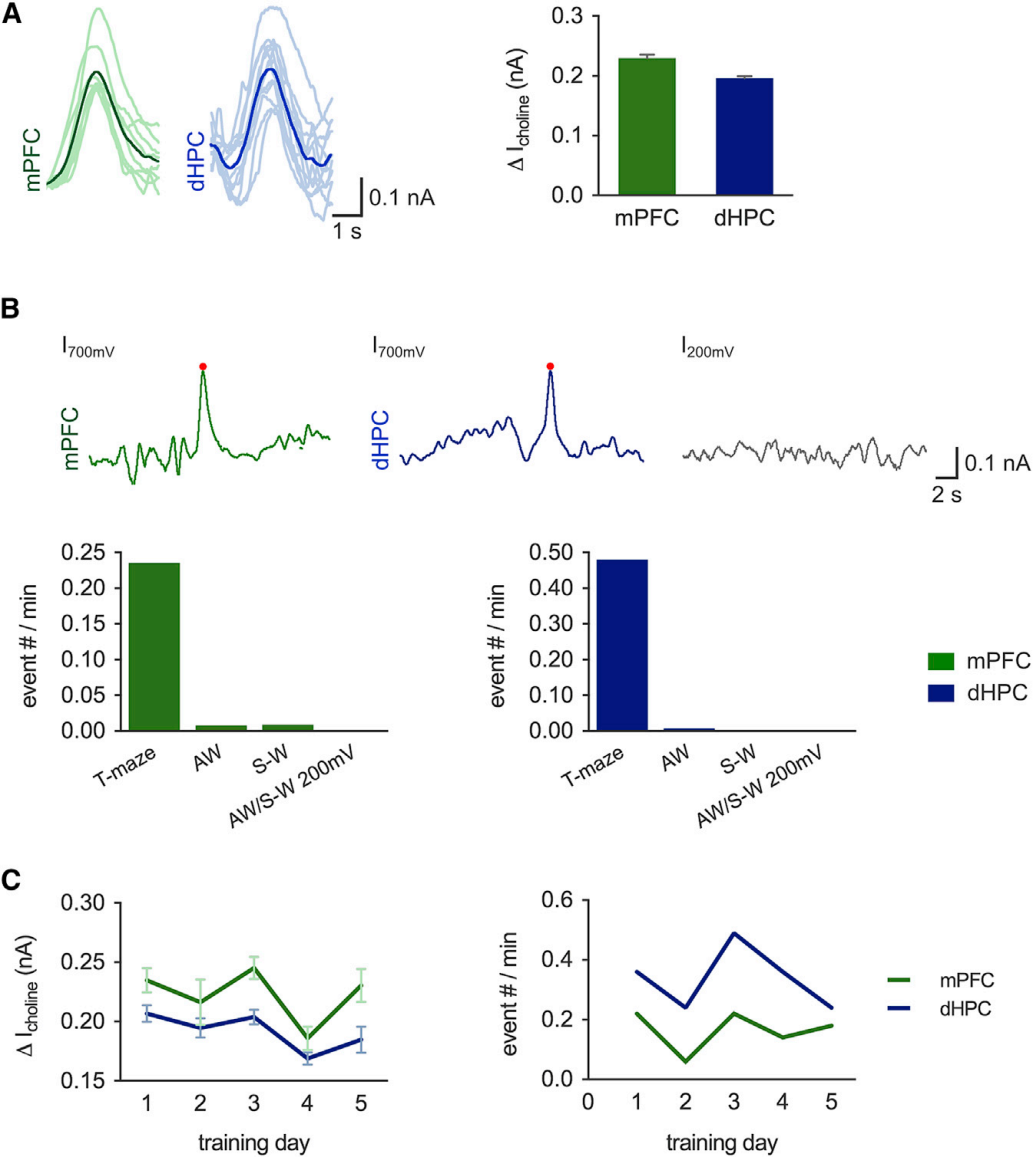
Phasic Release of Acetylcholine Occurs Predominantly during a Spatial Memory Task (A) Left: phasic acetylcholine release recorded in the mPFC and dHPC. Example individual release events (light traces) and average release events (dark traces). Right: average choline current amplitude for phasic release events (ns = 224 and 462 for the mPFC and dHPC, respectively, from 6 mice). (B) Example traces recorded at potentials of +700 mV and +200 mV. Detected phasic acetylcholine events are indicated by red dots. Phasic acetylcholine release events occurred almost exclusively during training on a spatial memory task. AW, active wakefulness; S-W, sleep-wake cycle. (C) The amplitude and frequency of phasic acetylcholine release events during maze training were constant across consecutive training days in both the mPFC and dHPC (n = 6 mice). Data are indicated as mean ± SEM.

Phasic acetylcholine release events were detected using a template-matching procedure followed by application of an event detection threshold of 3 SDs of the noise distribution and validated using recordings performed with an applied biosensor potential of +200 mV, where acetylcholine release is not detected (Figures 4A and 4B). Phasic acetylcholine release events with kinetic profiles similar to those from previous reports (Parikh et al., 2007) were found in both the mPFC (n = 224 from six animals) and dHPC (n = 462 from six animals), almost exclusively during maze training (Figures 4A and 4B) superimposed over tonic acetylcholine release (Figure 1B). Conversely, they were virtually absent during active wakefulness in the homecage immediately post-maze (Figure 4B), when the animals were still highly active (Figure 1B), or during sleep-wake cycles in the homecage (Figure 4B). The frequency and amplitude of phasic acetylcholine release events were consistent across consecutive training days, demonstrating an independence from task familiarity and performance (Figure 4C). Phasic acetylcholine release events in the mPFC and dHPC are, therefore, preferentially evoked during performance of a cognitive task, but their amplitude and overall frequency are independent of task performance.

**Figure 5:**
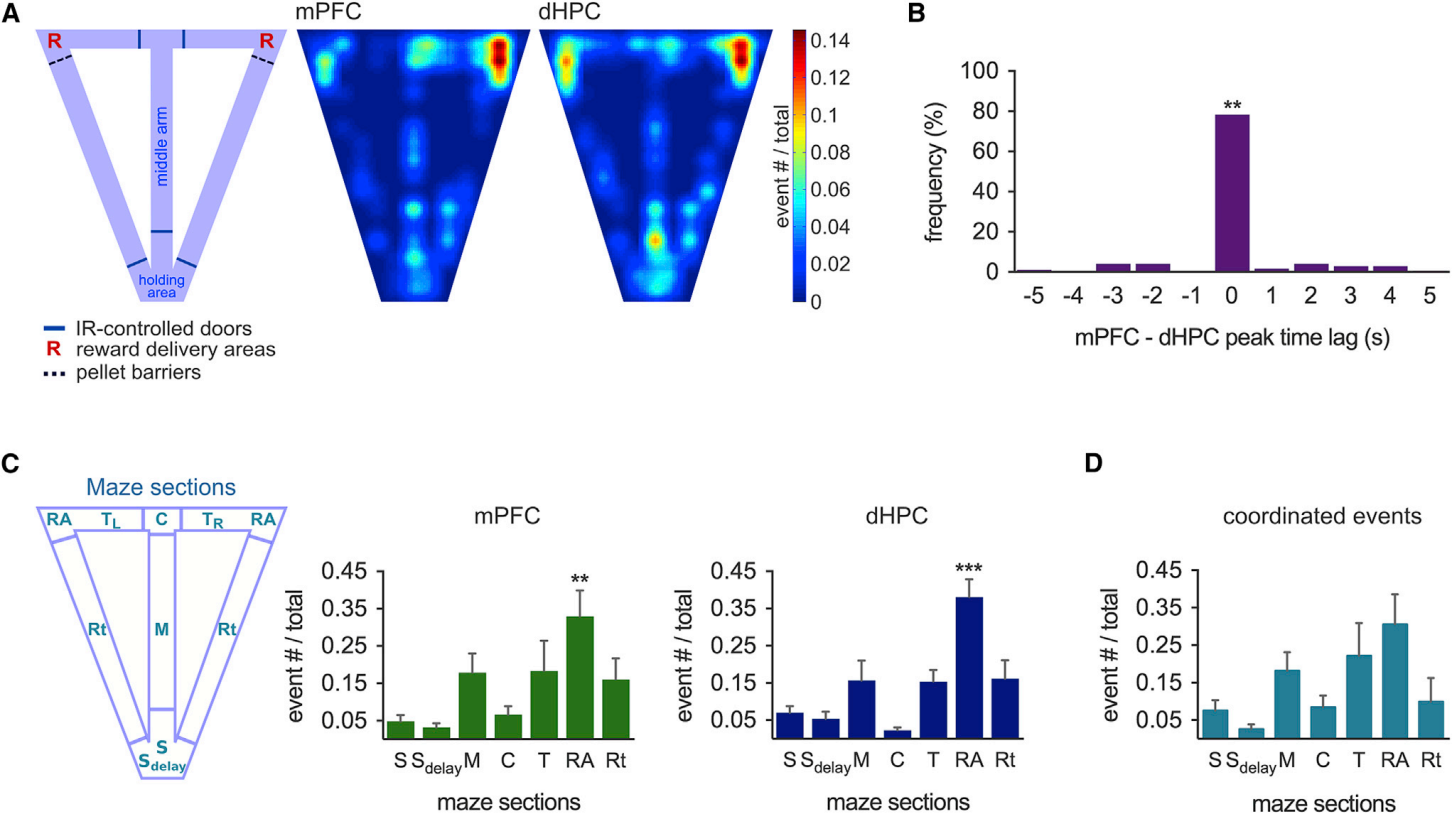
Phasic Release of Acetylcholine Is Associated with the Reward Location (A) Frequency distribution maps showing location of phasic acetylcholine release events during performance of a T-maze spatial memory task. IR, infrared. (B) Frequency distribution of phasic acetylcholine transients coordinated between the mPFC and dHPC within a 5-s time window (p < 0.01, compared to the probability of chance coordination within 1 s). (C) A higher incidence of phasic acetylcholine transients was detected in the mPFC and dHPC when animals were located in the reward-delivery area (n = 6 mice; p < 0.05 for both mPFC and dHPC, ANOVA with Dunnett post hoc correction). The distribution of events was similar in the mPFC and dHPC. Legend: S_delay_, 5-s delay holding area; S, holding area (trial start); M, middle arm; C, choice point; T_L/R_, forced-choice, left or right turn; RA, reward areas, left and right; Rt, return arms, left and right. (D) Coordinated phasic release events did not show a preference for any maze section (n = 6 mice; p > 0.05 for comparison with both the mPFC and dHPC, ANOVA with Dunnett post hoc correction). Data are indicated as mean ± SEM. ^∗∗^p < 0.01; ^∗∗∗^p < 0.001.

**Figure 6:**
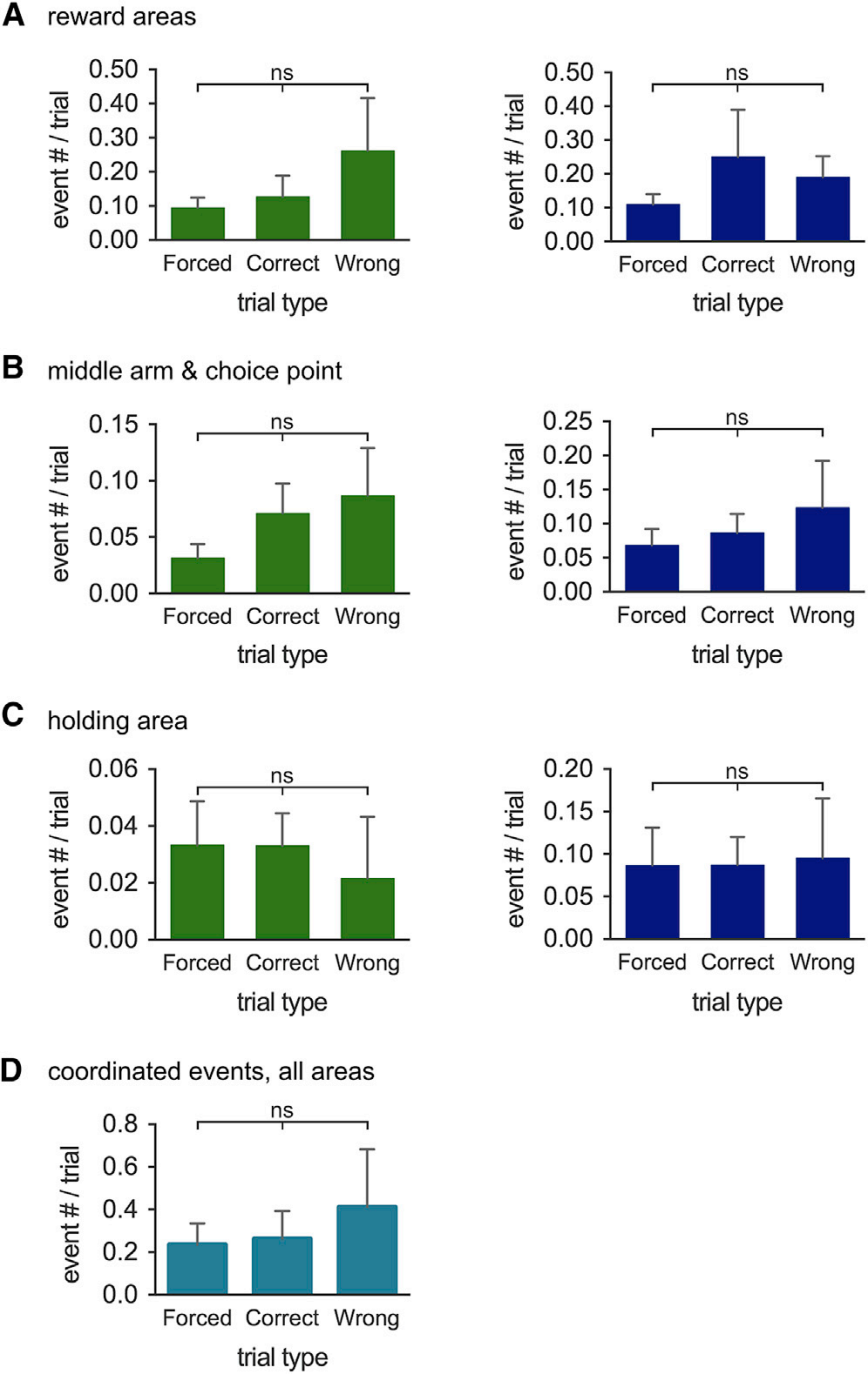
Phasic Release of Acetylcholine Is Independent of Trial Outcome (A–C) The frequency of phasic acetylcholine release events occurring in the reward location (A), middle arm and choice point (B), or holding area (C) for forced, correct-choice, and wrong-choice trials in the mPFC and dHPC. (D) Regardless of the maze section, the number of coordinated phasic events was comparable across trial types; n = 6 mice, all pairwise comparisons, ns (p > 0.05), ANOVA with Tukey HSD post hoc correction. Data are indicated as mean ± SEM.

We next tested when and where phasic acetylcholine release occurs during training on the T-maze spatial working memory task. Phasic acetylcholine release in both the mPFC and dHPC was strongly localized to the reward delivery areas, compared to other maze regions (Figures 5A and 5C; p < 0.05), even though animals spent similar amounts of time (and, therefore, pixel dwell time) in reward delivery areas, compared to the holding area or return arms. Importantly, phasic events showed high levels of coordination between the two brain regions. Of the total number of 224 events detected in the mPFC and 462 in the dHPC, 170 phasic transients in each region occurred within 5 s of an event in the other brain region (i.e., 75.9% of events in the mPFC and 36.8% of events in the dHPC), with 134 (78.4%) of these occurring within 1 s (Figure 5B; p < 0.01, compared to the probability of chance coordination). Coordinated phasic events were subsequently defined as occurring within a time window of 5 s and were found to occur without a preference for any given maze section (Figure 5D). There was no difference in incidence in the reward areas between right- and left-turn trials for the mPFC or dHPC (p > 0.05 in each case) and comparable incidence of phasic acetylcholine release in the reward delivery areas between forced-, correct-, and wrong-choice trials (when the animals received no reward) (Figure 6A). If only the largest phasic acetylcholine release events were considered (>0.2 nA; n = 65 for mPFC and n = 69 for dHPC), these were also preferentially localized to the reward areas and, again, not contingent on successful trial outcome (Figures S4A and S4B). The occurrence of phasic acetylcholine release events in reward delivery areas independent of reward delivery supports a role for phasic acetylcholine release in place-reward association rather than reward per se.

In rodents, the mPFC and dHPC show a transient coherence in theta frequency oscillations as they approach the choice point on the middle arm of the T-maze, which is thought to be important for task performance (Jones and Wilson, 2005, Kucewicz et al., 2011). Therefore, we analyzed the phasic acetylcholine events that occurred while the mice were on the middle arm or at the choice point of the maze to determine whether there was any correlation with trial outcome. We found that phasic acetylcholine release events that occurred on the middle arm or at the choice point occurred in the mPFC and dHPC with equal frequency during forced-, correct-, or wrong-choice trials (Figure 6B). This was also true for phasic acetylcholine release events that occurred in the holding area before commencing a trial (Figure 6C). Analysis of coordinated acetylcholine release revealed that there was no difference in the number of coordinated phasic events occurring during forced-, correct-, and wrong-choice trials (Figure 6D). These observations suggest that phasic acetylcholine release events are not the primary driver of enhanced theta coherence at choice points during successful performance on the spatial working memory task.

## Discussion

In this study, we made continuous recordings of acetylcholine release dynamics across a wide temporal range and simultaneously in two brain regions, the mPFC and dHPC. We confirmed that acetylcholine release can be classified into phasic and tonic modes that occur on distinct timescales and perform different roles (Sarter et al., 2009). We found that the two modes of transmission are not unique to the neocortex and also occur in the hippocampus. Tonic release was associated with arousal and the transition between specific vigilance states, whereas phasic release only occurred during behavior with the highest levels of arousal, i.e., while performing a cognitive task, where it occurred preferentially at the reward delivery locations. Surprisingly, both modes of transmission were coordinated between the mPFC and dHPC, indicating a brain-wide cholinergic signal.

Transitions into REM sleep or high-arousal states such as wakefulness are characterized by a switch from low-frequency oscillations to high-frequency oscillations or desynchronized neuronal networks that are also a feature of selective attention (Hasselmo and Sarter, 2011) and the selection of salient information relevant to reinforcement cues such as reward or punishment (Hangya et al., 2015, Lovett-Barron et al., 2014). This is thought to occur by increasing the signal-to-noise ratio of sensory input in primary sensory cortices (Chen et al., 2015, Eggermann et al., 2014, Fu et al., 2014, Pinto et al., 2013). It is proposed that a common mechanism underlying these states is increased acetylcholine release (Harris and Thiele, 2011). Using continuous recordings across multiple brain states, we aimed to determine whether acetylcholine release correlates with these behavioral states. We show that acetylcholine release is strongly associated with high-arousal states and location of the animal in the reward delivery area on a T-maze spatial working memory task, suggesting that acetylcholine is released in response to reward or the expectation of reward. These observations broadly support a role for cholinergic input for the desynchronization of networks during increases in arousal or attentional states.

Specifically, we show a conditional and coordinated increase in tonic acetylcholine during REM sleep, which suggests that acetylcholine may be preparing mPFC and dHPC networks simultaneously for wakefulness (Jones, 2004) and the enhanced vigilance required for the performance of tasks requiring sustained attention (Paolone et al., 2012). Although it is likely that GABAergic projections control switching between behavioral states (Anaclet et al., 2015, Chen et al., 2016), cholinergic inputs play the role in network state modulation (Fisahn et al., 1998, Lee et al., 1994). In addition, our continuous recordings of acetylcholine release with high temporal resolution show that REM sleep cannot be classified as a single homogeneous state and that REM epochs occurring in the middle of NREM epochs may be performing roles different from those occurring immediately before wakefulness. The underlying mechanism for REM epoch heterogeneity may result from the complexity of brainstem circuitry controlling REM sleep initiation and maintenance (Saper et al., 2010). The core finding that the magnitude of tonic acetylcholine release during REM is predictive of subsequent wakefulness demonstrates a previously unappreciated role for acetylcholine release during REM sleep.

REM sleep is proposed to create an environment to facilitate plasticity processes that create a generalized downregulation of synaptic strength (Grosmark et al., 2012, Tononi and Cirelli, 2014), while synapses are upregulated specifically by the reactivation of neuronal firing sequences experienced during salient events found in REM and NREM sleep episodes (Atherton et al., 2015, Lee and Wilson, 2002, Louie and Wilson, 2001). Our findings for the release of acetylcholine only during some periods of REM sleep, and not during NREM sleep, suggest that acetylcholine may enable the dual processes of generalized synaptic downregulation and specific synaptic potentiation to occur in different phases of sleep and, therefore, facilitate efficient memory consolidation.

The importance of phasic acetylcholine release to attention and cue detection has been demonstrated by the lack of cue detection in the absence of phasic cholinergic events in the prefrontal cortex (Gritton et al., 2016, Parikh et al., 2007) and a reduction in attentional performance in animals with reduced cholinergic innervation, which may be rescued by cholinergic agonists (Paolone et al., 2013). Further evidence suggests that phasic acetylcholine release in the mPFC shifts the behavioral state from cue monitoring to activation of response rules and subsequent responses (Howe et al., 2013). However, this view is challenged by data showing that BF non*-*cholinergic, but not cholinergic, neuron activity is correlated with performance accuracy (Hangya et al., 2015). We found that coordinated phasic acetylcholine release between the mPFC and dHPC occurs only during maze performance. This suggests that phasic acetylcholine release is important for task performance and shows that phasic release is not limited spatially to the mPFC but also occurs in the dHPC. In our study, the occurrence of phasic acetylcholine release events in the reward-delivery areas, regardless of reward delivery and independent of successful task completion, indicates a response to reward or the expectation of reward. This supports previous theories for the role of acetylcholine release as a reinforcement signal to guide learned behavior in response to salient cues and the dependence of cholinergic activation on outcome expectation (Hangya et al., 2015), thus suggesting a role for coordinated phasic release of acetylcholine in the mPFC and dHPC for the accessing of retained place-reward associations (internal cues) necessary for successful task completion. Thus, the coordinated phasic release of acetylcholine may be important for the processing of both externally and internally stored cues relevant to salient events (Baddeley, 2003), enabling the assessment of uncertainty (Yu and Dayan, 2005). Furthermore, the release of acetylcholine in the mPFC and dHPC in the same spatial locations implies that place-reward association requires coordinated reorganization of network function in these interconnected structures.

The PFC and HPC are both required for the successful learning of spatial working memory tasks, including delayed non-match to place tasks such as the T-maze task used in this study. The direct synaptic connection between the ventral HPC and mPFC is required for the acquisition phase of working memory potentially by synchronizing the two brain areas within the gamma frequency range (Spellman et al., 2015). Equally, synchronization of the mPFC and dHPC within the theta frequency range at the choice point and, therefore, retrieval phase of the task is also important (Jones and Wilson, 2005, Kucewicz et al., 2011) and is disrupted in an animal model of schizophrenia with poor working memory performance (Sigurdsson et al., 2010). Acetylcholine release amplifies both theta and gamma frequency oscillations (Fisahn et al., 1998, Lee et al., 1994); therefore, its coordinated release in the mPFC and HPC is predicted to contribute to the transient increases in mPFC-HPC theta and gamma coherence that underlie successful trial performance. Although our experiments are not designed to test this hypothesis directly, our observation that phasic release of acetylcholine is coordinated in the mPFC and dHPC suggests that it may play a role in controlling mPFC-HPC theta and gamma coherence.

In this study, we make the first simultaneous recordings of acetylcholine release in multiple brain regions at a temporal resolution less than 1 s. One of the most striking findings is that acetylcholine release has a remarkably similar temporal profile in the mPFC and dHPC, suggesting a coordinated action of the BF and MS-DBB cholinergic signaling pathways for both tonic and phasic release. This aligns with data showing behavioral state-dependent firing of central cholinergic neurons across the medial septum and nucleus basalis (Hangya et al., 2015). The circuit mechanisms underlying coordinated cholinergic activity may arise from inter-nuclei connectivity (Zaborszky and Duque, 2000) where glutamatergic neurons are known to excite cholinergic neurons to promote wakefulness (Xu et al., 2015). Thus, our data support a model where synchronous activation of distinct central cholinergic nuclei with non-overlapping projections enables this neuromodulatory system to broadcast a unified, highly precise signal to multiple areas of the brain simultaneously engaged in information processing and behavioral task performance. However, there may still be instances where selective activation of discrete nuclei and release of acetylcholine in distinct regions are important (Apparsundaram et al., 2005, Bloem et al., 2014, Martinez and Sarter, 2004).

At the cellular level, the wide range of acetylcholine receptor subtypes, with distinct affinities, desensitization characteristics, and cellular locations, is likely to be differentially engaged by tonic and phasic modes of cholinergic transmission. It is tempting to speculate that higher affinity muscarinic receptors integrate tonic acetylcholine release, whereas lower affinity desensitizing nicotinic receptors respond preferentially to phasic release, which may enable distinct populations of neurons to respond appropriately according to specific cognitive operations. For example, recruitment of disinhibitory circuits via nicotinic receptors has been shown to modulate cortical arousal and to drive reinforcement responses in cortical neurons (Letzkus et al., 2011, Pi et al., 2013), whereas muscarinic receptor activation opens a window for the induction of NMDA-receptor-dependent synaptic plasticity and associative learning (Anagnostaras et al., 2003, Buchanan et al., 2010, Isaac et al., 2009).

Overall, the coordinated release of acetylcholine presented in this study suggests a model where cholinergic signaling supports brain-wide state transitions by enabling the processing of salient information either as phasic release to encode reinforcement cues or as tonic release to encode arousal.

## Experimental Procedures

### Ethics Statement

All procedures were conducted in accordance with the UK Animals (Scientific Procedures) Act 1986 and the Eli Lilly UK Ethics Committee.

### Subjects and Housing Conditions

Male 6-week-old C57BL/6J mice were housed in standard housing conditions with five mice per cage on a normal light/dark cycle.

### Choline Biosensors

All biosensor preparation and calibration was performed in the BioAnalytics Laboratory at the Department of Chemistry, Maynooth University, Ireland (Baker et al., 2015). In brief, both ends of Teflon-coated Pt/Ir (90%/10%) cylinder electrodes (125-μm bare diameter, 175-μm coated diameter) were stripped of the Teflon insulation. One end was coated with a layer of electropolymerized ortho-phenylenediamine (PPD). The PPD-modified electrode was then dipped into methyl methacrylate and cellulose acetate solutions and then sequentially dipped into ChOx, BSA, glutaraldehyde, and polyethyleneimine using a dip adsorption method. The process was repeated ten times, with each layer being allowed to dry for 5 min, producing a PPD-polymer-composite (PC)/ChOx-modified electrode (Pt/PPD-PC/ChOx/PC) (Baker et al., 2015). Pt-based polymer enzyme composite biosensors designed with a large cylindrical geometry increase the target analyte (H_2_O_2_) signal relative to the fundamental noise of the potentiostat amplifiers. Repeated layering (ten times) of the polymer-composite coating embedded with ChOx further increases biosensor sensitivity (375 pA/μM; Figure S2). The well-characterized chemical rejection underlayer (PPD) (Lowry et al., 1998, Lowry and O’Neill, 1994) makes up the interference rejection layer making the biosensors highly selective for choline.

Choline microelectrochemical biosensors monitor extracellular choline by detecting the oxidation of H_2_O_2_, a by-product of choline breakdown by the ChOx enzyme embedded in the polymer coating. H_2_O_2_ oxidation is the current generating electrochemical step (Figure S2A). Changes in the current produced by the electrochemical oxidation of H_2_O_2_ are, therefore, directly proportional to the local extracellular tissue concentration of choline (Baker et al., 2015). Biosensor fabrication with permselective polymers also addresses selectivity issues associated with the enzyme mediator O_2_, and access to the electrode surface by electroactive agents or neurotransmitters (Dixon et al., 2002, Lowry et al., 1998, Lowry and O’Neill, 1994).

Before implantation, biosensors were calibrated in vitro in a standard electrochemical cell. Calibrations were performed in 20 mL of PBS solution, pH 7.4, where the concentration of choline was increased from 0 to 3 mM. The lower limit of detection of these biosensors was 100 nM. Biosensors were chosen for implantation if the measured current values from the saturated solutions were not significantly different from the average (Figure S2C). The ratio between the measured choline current (in nanoamperes) and the corresponding biosensor sensitivity value (in nanoamperes per micromolar) provided an estimate of extracellular acetylcholine concentrations.

### Surgical Implantation of Choline Biosensors

Choline biosensors were implanted in the mPFC and the dHPC under isoflurane anesthesia. An LFP electrode was implanted in the CA1 pyramidal layer of the dHPC (Figure S1).

### In Vivo Constant Potential Amperometry and LFP Recordings

Each head-mounted six-pin pedestal was tethered to a low-noise, four-channel potentiostat (EA164 QuadStat, eDAQ) and to a DP-301 differential amplifier (Warner Instruments) via a flexible six-core cable mounted through a swivel in the ceiling of the recording chamber to allow free movement of the animals throughout the recording cages.

Changes in extracellular tissue choline concentration were measured using constant potential amperometry (+700 mV). Day-matched homecage control recordings were performed at +200 mV, at which the contribution of the current generated by H_2_O_2_ oxidation at the sensor surface is minimized (Figure S2D). After application of a potential to the biosensors, the signal was allowed to settle for approximately 24 hr. Data were then collected continuously for 12 hr during the light phase over a period of 5 consecutive days.

Hippocampal local field potentials were recorded using differential amplification, low-pass (1-kHz) and high-pass (0.1-Hz) filters, and an output gain of 1,000.

A 50-Hz low-pass digital filter was applied post hoc to both the chemical and electrical signals. Choline and LFP data were digitized with a 16-channel eCorder unit (ED1621, eDAQ) and acquired with Chart (v5.5.18, eDAQ). All data were recorded at a 1-kHz sampling rate.

### Randomized Forced Alternation T-Maze Test

Animals that recovered their pre-surgery weight were food restricted overnight and tested on an automated T-maze the following morning. All mice were tested at the same time of day over the 5 consecutive training days (Figure 3A).

Entry of the mice into specific areas of the maze was detected using infrared beam breaks that automatically controlled the maze protocol. Rewards were delivered by two pellet dispensers located at the end of each reward arm. An infrared video camera recorded animal location during maze performance and classification of trials.

Each trial on the maze comprised two stages: a sample (forced) phase and a test (choice) phase (Figure 3B). A 5-s delay was applied between sample and test phases (Ainge et al., 2007). The average time, in seconds, taken for each mouse to travel between infrared beams on the central and choice arms during choice trials was defined as the average choice latency time. Left/right allocations for the sample and choice runs were pseudo-randomized, with no more than three consecutive sample runs to the same side.

Training on the task was not performed before the beginning of behavioral testing. During testing, animals were allowed to run up to 20 trials in a 60-min period. At the end of each session, animals were returned to their chambers, with ad libitum access to food and water.

### Histology

At the end of the experiments, animals were deeply anesthetized with pentobarbital and perfused transcardially with 10% buffered paraformaldehyde (PFA). To confirm biosensor electrode placement, serial 50-μm mPFC and dHPC sections were cut in the coronal plane using a cryostat.

Gliosis around the biosensors was assessed by immunostainings against Iba-1 and glial fibrillary acidic protein (GFAP) (Figure S1C). 6-μm-thick whole-brain coronal sections were incubated in primary rabbit anti-GFAP (1:4,000, AR020-5R, Biogenex) or primary rabbit anti-Iba-1 (1:600, 019-19741, Wako Pure Chemicals Industries) antibodies for 60 min at room temperature, followed by secondary biotinylated goat anti-rabbit antibody (1:200, BA-1000, Vector Laboratories) for 30 min at room temperature. Antibody labeling was achieved with ABC-horseradish peroxidase conjugate and 3,3′-diaminobenzidine chromagen (1:30). Counterstaining was performed in hematoxylin (1:1). All slides were imaged with an Aperio digital slice scanning system (Leica).

### Locomotor Activity Analysis

Locomotor activity was monitored continuously using infrared cameras and analyzed using a script from NIH Image as previously described (Richmond et al., 1998). In these experiments, a difference of less than 50 pixels resulted in a “no-movement,” score and the mouse was judged to be sleeping.

### Sleep Scoring

Arousal states were determined using the automated sleep scoring algorithm based on SCORE (Van Gelder et al., 1991). Short periods of wakefulness with low locomotor activity (between 50 and 200 Δ pixels) that occurred between sleep cycles were labeled as quiet wakefulness. For analysis purposes, wake and theta-dominated wakefulness were combined and designated as the active wake (AW) state.

To plot changes in theta frequency power, raw LFP data were band-pass filtered between 0.7 Hz and 30 Hz and downsampled to 100 Hz. Fourier power analysis was performed using the Chronux tool box. The ratio of the power in the theta (6–12 Hz) frequency band was calculated with a moving window (5 s, 0.5-s step) and z-normalized.

### Data Analysis

#### Behavior-Dependent Event-Triggered Analysis

In vivo amperometry data were analyzed using custom written MATLAB scripts. Data were low-pass filtered at 2 Hz and smoothed with a sliding window (width, 1 s). For each recording session in the homecage, three behavioral states were defined based on the scored data— active wake, REM, and NREM. REM epochs were only included in the analysis if preceded by a minimum of 20 s (two consecutive 10-s bouts) of NREM. Each behavioral state was further split into a series of behavioral sequences (see Table 1).

#### Phasic Transient Analysis

Detection of phasic choline transients was done using the ClampFit template-matching tool (Molecular Devices) (Clements and Bekkers, 1997). Template waveforms were created for each implanted biosensor by averaging three to six large events detected by visual inspection. Events that were part of equal and opposite positive and negative going deflections were deemed non-biological and excluded. Events that were smaller than three times the average SD of the raw data (3σ) for each animal were also excluded. Average SDs were similar during T-maze training or in the homecage with the sensor potential set at +700 mV or +200 mV and were consistent between mice (0.06 ± 0.004 nA for mPFC [n = 30], and 0.06 ± 0.004 nA for dHPC [n = 32] for six mice; all pairwise comparisons, not significant [ns], by ANOVA with Tukey honestly significant difference [HSD] post hoc correction). A comparison of the amplitude frequency distribution of events detected at biosensor potentials of +200 mV and +700 mV both in the homecage and on the maze revealed that a 3σ amplitude threshold excluded almost all template-matched events occurring at +200 mV and, therefore, not choline mediated (Figure 4B).

To calculate the proportion of transients that occurred in each maze section, the data were normalized to the total number of transients detected in each brain region and plotted as a color plot smoothed with a 2D Gaussian low-pass filter.

### Statistical Analysis

Statistical significance and normality tests were performed using tests in SPSS (v23.0.0.2, IBM). Where data did not pass the Levene’s test for equal variance between groups, one-way Welch’s ANOVA was used for all multiple comparisons tests with Games-Howell post hoc adjustment. Otherwise, a standard one-way ANOVA was used with Tukey HSD or Dunnett post hoc adjustment or a two-tailed paired t test for within-animal comparison of +700 mV to +200 mV REM transients. Two-tailed Mann-Whitney tests were used for comparisons between two independent groups. Unless otherwise stated, data are reported as means ± SEM; ns denotes p > 0.05, ^∗^p < 0.05, ^∗∗^p < 0.01, and ^∗∗∗^p < 0.001.

## Author Contributions

Conceptualization, L.M.T.-G.R., J.T.R.I., and J.R.M.; Methodology, K.L.B., J.P.L., G.G., and K.G.P.; Software, M.W.C. and P.J.K.; Investigation, L.M.T.-G.R. and K.L.B.; Resources, K.L.B. and J.P.L.; Writing, L.M.T.-G.R., G.G., J.T.R.I., J.P.L., and J.R.M.; Visualization, L.M.T.-G.R. and J.R.M.; Supervision, G.G., K.G.P., J.T.R.I., J.P.L., and J.R.M.; Funding Acquisition, J.T.R.I., J.P.L., and J.R.M.

## Figures and Tables

**Table 1:** List of Behavioral Sequences Defined for Behavior-Dependent Event-Triggered Analysis

Behavioral State	Minimum Epoch Length (s)	Preceding State	Minimum Epoch Length (s)	Following State	Minimum Epoch Length (s)
(REM-) AW^a^	600	REM	20	n/a	n/a
(NREM-) AW^a^	600	NREM	20	n/a	n/a
REM^a^ (-Wake)	20	NREM	20	Wake	10
REM^a^ (-NREM)	10	NREM	20	NREM	60
NREM^a^ (-Wake)	60	n/a	n/a	Wake	10

n/a, not applicable.

a Main behavioral state: REM, AW (active wakefulness), or NREM (non-REM).

## Supplemental Information

Document S1. Supplemental Experimental Procedures, Figures S1–S4, and Table S1

Document S2. Article plus Supplemental Information
