# Supplementary material for: Coordinated Acetylcholine Release in Prefrontal Cortex and Hippocampus Is Associated with Arousal and Reward on Distinct Timescales
Source: Cell Rep. 2017 Jan 24;18(4):905–17. doi: 10.1016/j.celrep.2016.12.085 (PMC5289927; doi:10.1016/j.celrep.2016.12.085)
Supplement: Document S1. Supplemental Experimental Procedures, Figures S1–S4, and Table S1 [file mmc1.pdf]

Cell Reports, Volume *18*

Supplemental Information

Coordinated Acetylcholine Release in Prefrontal Cortex and Hippocampus Is Associated with Arousal and Reward on Distinct Timescales

Leonor M. Teles-Grilo Ruivo, Keeley L. Baker, Michael W. Conway, Peter J. Kinsley, Gary Gilmour, Keith G. Phillips, John T.R. Isaac, John P. Lowry, and Jack R. Mellor

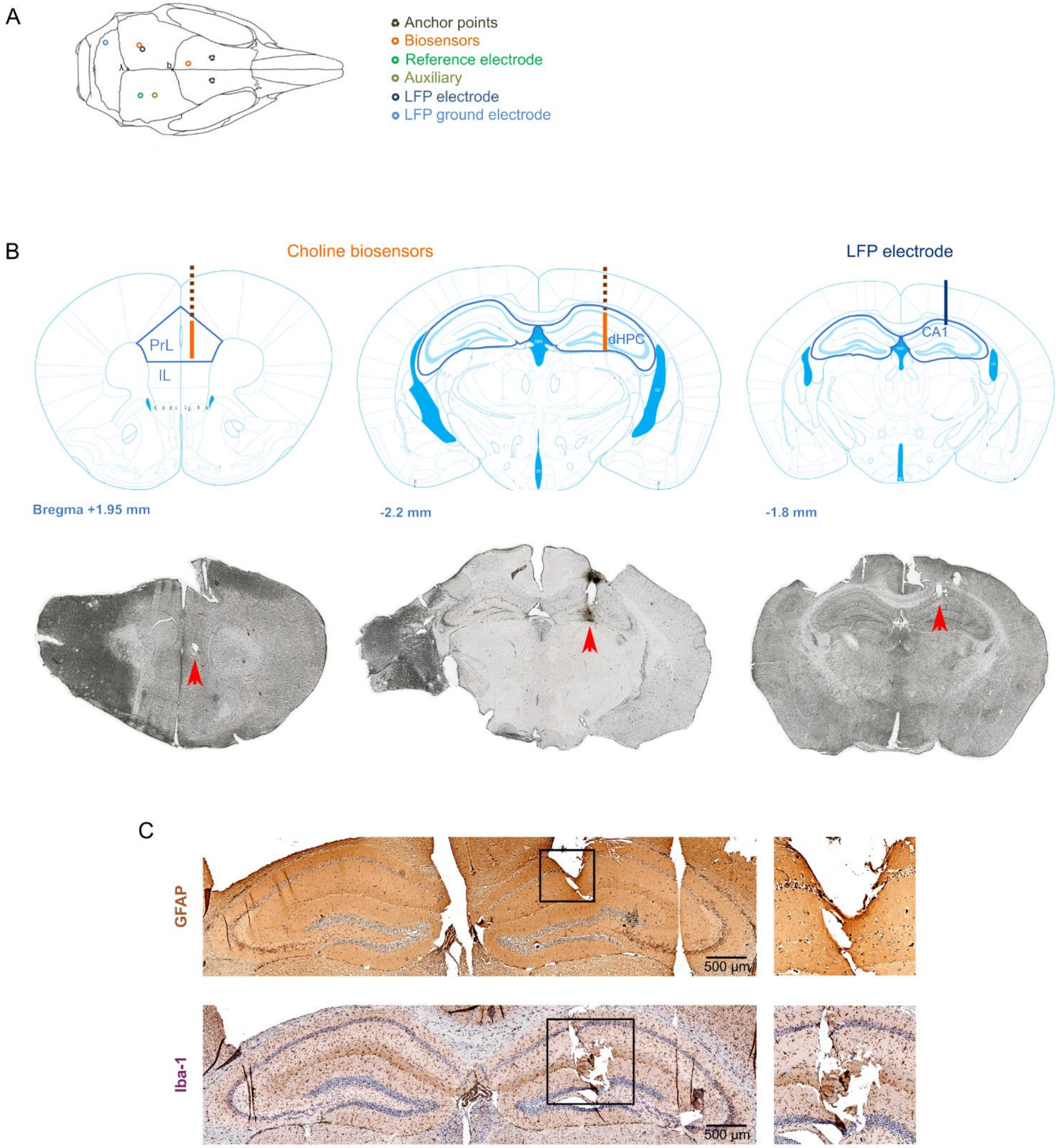

Figure S1. Biosensor and electrode placement. Related to Experimental Procedures.
A. Electrode implant diagram. Choline biosensors (orange) were implanted in the mPFC and ipsilateral dHPC. Choline reference (bright green) and auxiliary (olive green) electrodes were implanted in the contralateral side and the LFP electrode (dark blue) as close to the hippocampal sensor as possible. Skull screws placed anteriorly and posteriorly to ensure head cap stability (brown). b – bregma; λ – lambda. **B.** Coronal plane figures of the mPFC and dHPC illustrating the stereotaxic coordinates followed to implant choline biosensors and LFP electrode and histological confirmation of electrode placement (red arrow heads). **C.** Iba-1 and GFAP immunohistochemistry to assess the extent of gliosis around the implanted probes. There was no exacerbated migration of microglia or astrocytes to the site of biosensor implantation.

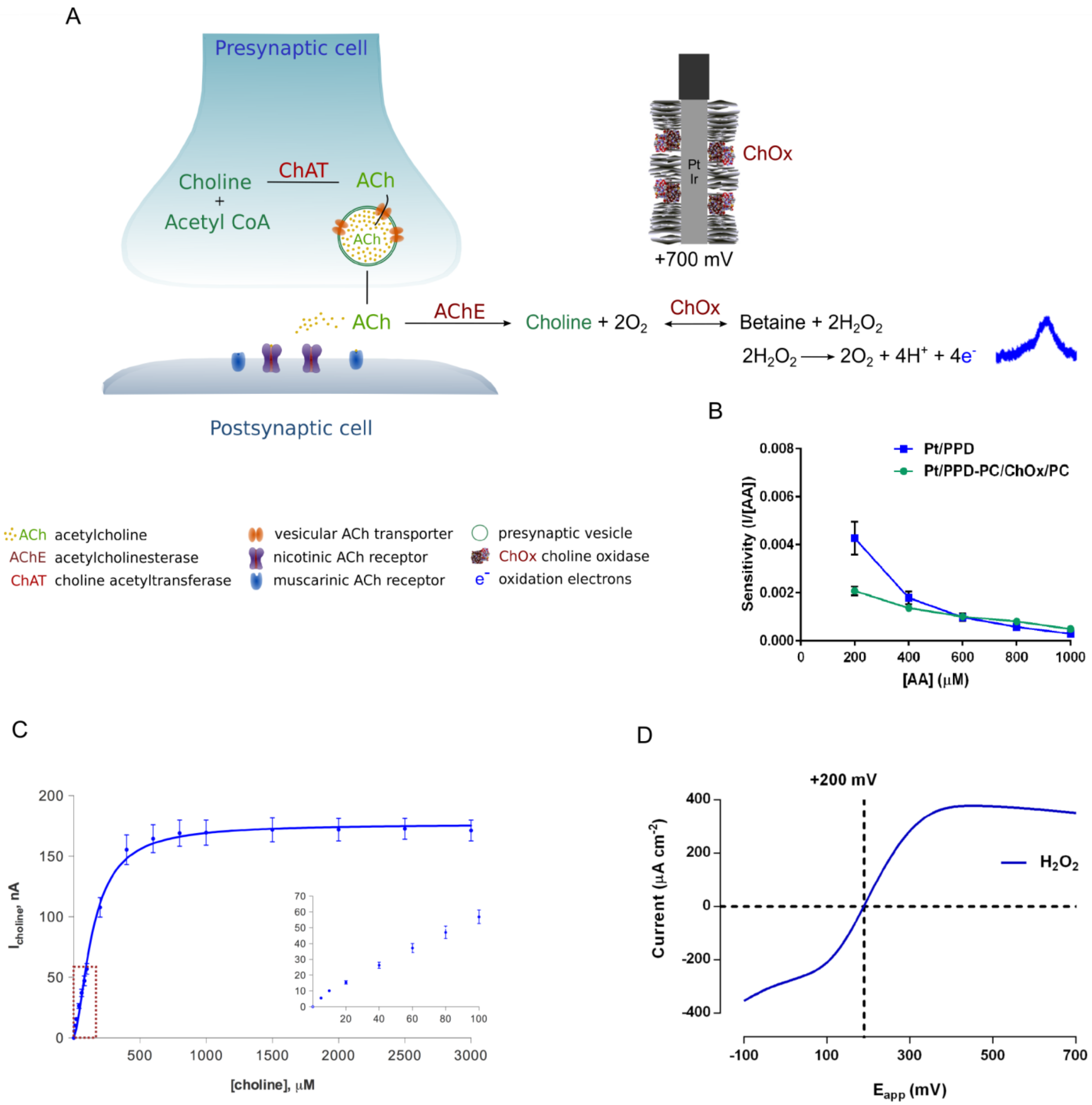

Figure S2. Choline biosensor design and application. Related to Experimental Procedures.
A. Acetylcholine is synthesized from choline and acetyl coenzyme A. The reaction takes place in axon terminals and is catalysed by the enzyme choline acetyltransferase. Acetylcholine is packaged into presynaptic vesicles by vesicular acetylcholine transporters and once it is released, it is quickly broken down by acetylcholinesterase. Choline biosensors detect the choline that results from the breakdown of synaptic acetylcholine release. Choline oxidase is embedded in the biosensor matrix and converts choline to betaine and hydrogen peroxide. Oxidation of H_2O_2 is the current generating electrochemical step. **B.** The sensitivity of Pt/PPD and Pt/PPD-PC/ChOx/PC sensors as a function of ascorbic acid (AA) concentration showing significant interference rejection (0.002 nA/µM *vs* 0.545 nA/µM at bare Pt) and steady-state saturation characteristics at physiological levels typical of PPD (Lowry and O'Neill, 1994). **C.** *In vitro* choline biosensor calibrations. Choline current saturates at high choline concentrations. Inset shows initial linear portion of the concentration-current relationship. **D.** Linear sweep voltammetry shows that the reversal potential for the H_2O_2 redox reaction at Pt is +200 mV indicating that at this potential choline cannot be detected.

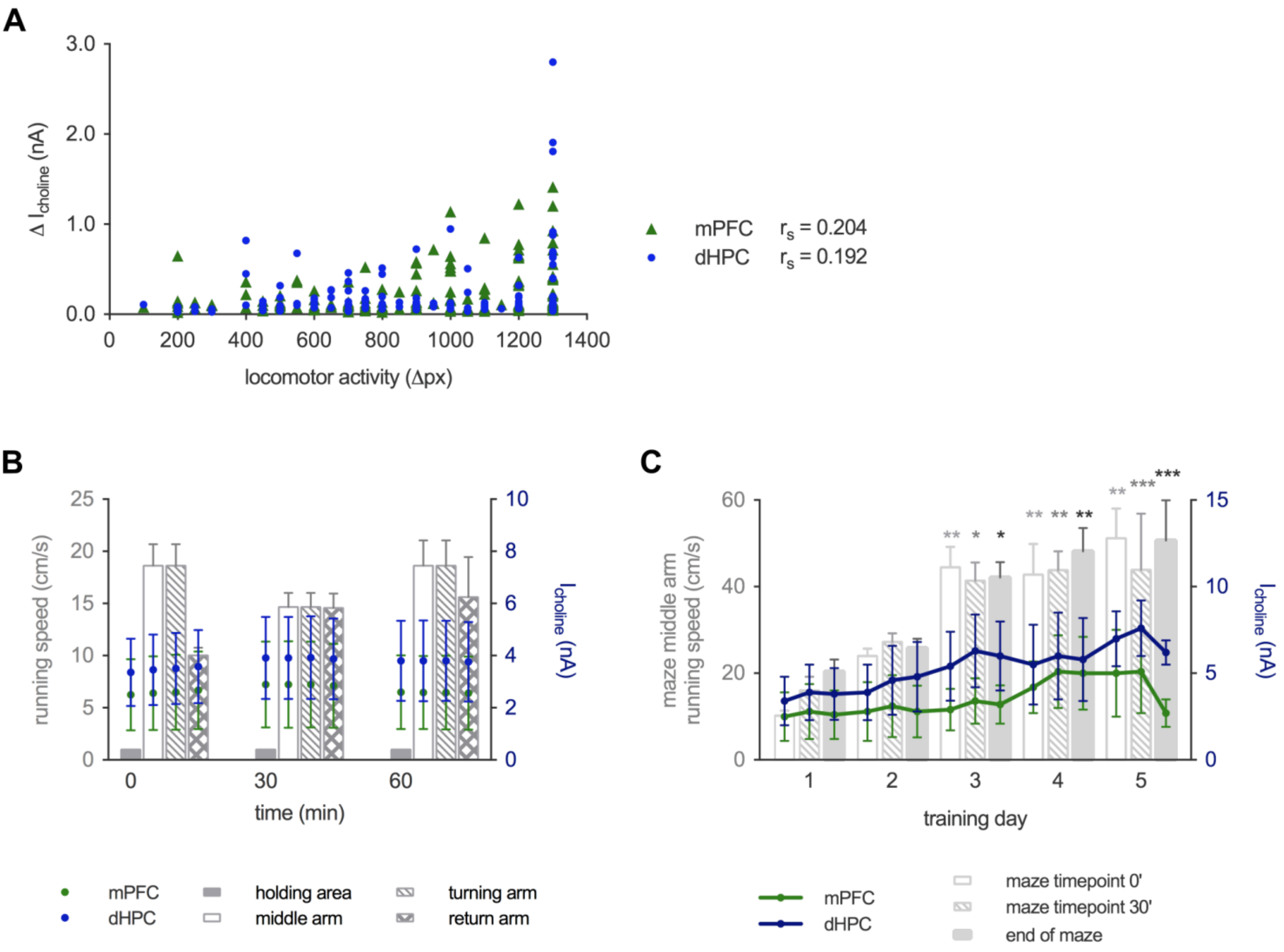

Figure S3. Tonic acetylcholine release is independent of locomotor activity levels. Related to Figure 1.
A. During periods of active wakefulness, tonic choline current was not strongly correlated with locomotor activity in the homecage (n = 159 periods in 6 animals, Spearman correlation). **B.** Running speed was consistent over the course of training sessions but varied according to each maze section. Tonic choline current did not vary according to maze section indicating no correlation between running speed and choline current. **C.** Running speed during maze performance increased significantly across training days without affecting tonic acetylcholine release (n = 6 mice). *, ** or *** denote pairwise comparisons with day 1, ANOVA with Dunnett posthoc correction.

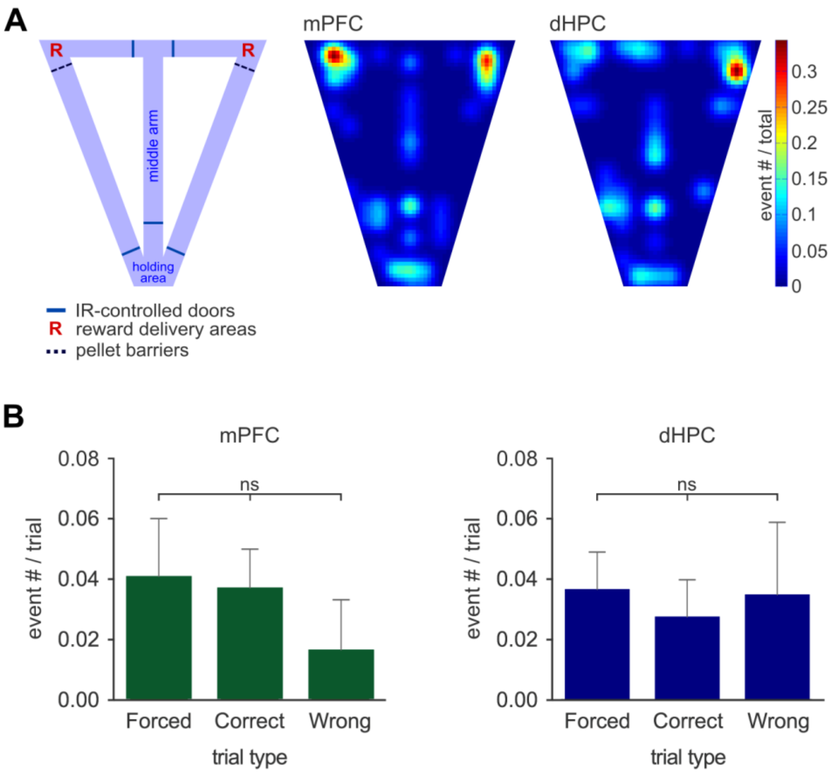

Figure S4. Phasic release of acetylcholine is associated with the reward location when only events >0.2nA are considered. Related to Figures 5 & 6.

A. Frequency distribution maps showing location of phasic acetylcholine release events during performance on a T-maze spatial memory task. **B.** The frequency of phasic acetylcholine release events occurring in the reward location for forced, correct- and wrong-choice trials. n = 6 mice, all pairwise comparisons n.s., ANOVA with Tukey HSD posthoc correction. Data shown as mean ± SEM.

Supplementary Experimental Procedures

Ethics statement

All procedures were conducted in accordance with the UK Animals Scientific Procedures Act (1986) and the Eli Lilly UK Ethics Committee.

Subjects and housing conditions

Male, 6-weeks old C57BL/6J mice were supplied by Charles River (Margate, UK) and housed at Eli Lilly (Windlesham, UK) in standard housing conditions: 5 mice per individually ventilated cages, normal light/dark cycle (lights on from 7:00 to 19:00), controlled temperature (21-22°C) and humidity (40-42%), *ad libitum* access to chow food pellets and water.

During the surgery recovery period, animals were habituated to handling, untethered exploration of the recording cages (3 hours/day), and to the sucrose pellets used as a reward in the T-maze task. One week following recovery, mice were singly housed in small rodent housing cages (1284, Tecniplast) modified to allow for continuous recordings with free movement and *ad libitum* access to chow food pellets and water until the beginning of behavioral training. Each cage was placed inside a light- and sound-attenuating chamber with temperature and humidity monitors, timer-controlled LEDs to maintain the standard 07:00 to 19:00 light phase, and an infra-red camera to record locomotor activity (LMA). Body weight was monitored daily.

A total of ten 7-weeks old C57BL/6J male mice weighing 19 – 23 g were used in this study. Mice that did not recover their pre-surgery weight were not included in the T-maze testing cohort. Data were excluded from the analysis if any one of the following criteria was met: incorrect placement of at least one of the biosensors or LFP electrode, poor LFP signal quality, poor choline signal stability. 6 mice met all the criteria required for data analysis.

Choline biosensors

All biosensor preparation and calibration was performed in the BioAnalytics Laboratory at the Department of Chemistry, Maynooth University, Ireland (Baker et al., 2015). In brief, both ends of Teflon®- coated Pt/Ir (90%/10%) cylinder electrodes (125 µm bare diameter, 175 µm coated diameter, Advent Research Materials) were stripped of the Teflon® insulation. One end was soldered into a gold clip (Fine Science Tools GmbH). The other end was coated with a layer of electropolymerised ortho-phenylenediamine (1,2-diaminobenzene (PPD), ≥98%, Sigma-Aldrich). The PPD-modified electrode was then dipped into methyl methacrylate (99%, Sigma-Aldrich) and cellulose acetate solutions, and then sequentially dipped into choline oxidase (ChOx; from *Alcaligenes sp.*, EC 232-840-0, Sigma-Aldrich), bovine serum albumin (fraction V from bovine plasma, Sigma-Aldrich), glutaraldehyde (Grade 1, 25%, Sigma-Aldrich), and polyethyleneimine (80% ethoxylated, Sigma-Aldrich) using a dip adsorption method. The process was repeated 10 times with each layer being allowed to dry for 5 minutes producing a PPD-polymer composite (PC)/ChOx-modified electrode (Pt/PPD-PC/ChOx/PC) (Baker et al., 2015). Pt-based polymer enzyme composite biosensors designed with a large cylindrical geometry increase the target analyte (H_2O_2) signal relative to the fundamental noise of the potentiostat amplifiers. Repeated (10) layering of the polymer-composite coating embedded with choline oxidase further increases biosensor sensitivity (375 pA/µM; Fig. S2). The well characterised chemical rejection underlayer (PPD) (Lowry et al., 1998; Lowry and O'Neill, 1994) makes up the interference rejection layer making the biosensors highly selective for choline.

Choline microelectrochemical biosensors monitor extracellular choline by detecting the oxidation of hydrogen peroxide (H_2O_2), a by-product of choline breakdown by the choline oxidase (ChOx) enzyme embedded in the polymer coating. Hydrogen peroxide oxidation is the current generating electrochemical step (Fig. S2A). Changes in the current produced by the electrochemical oxidation of H_2O_2 are therefore directly proportional to the local extracellular tissue concentration of choline (Baker et al., 2015). Biosensor fabrication with permselective polymers also addresses selectivity issues associated with the enzyme mediator O_2, and access to the electrode surface by electroactive agents or neurotransmitters (Dixon et al., 2002; Lowry et al., 1998; Lowry and O'Neill, 1994).

Before implantation, biosensors were calibrated *in vitro* in a standard electrochemical cell. Calibrations were performed in 20 ml of PBS solution, pH 7.4, where the concentration of choline was increased from 0 to 3 mM. The lower limit of detection of these biosensors was 100 nM. Biosensors were chosen for implantation if the measured current values from the saturated solutions were not significantly different from the average (Fig.

S2C). The ratio between the measured choline current (in nA) and the corresponding biosensor sensitivity value (in nA/μM) provided an estimate of extracellular acetylcholine concentrations.

Surgical implantation of choline biosensors

Choline biosensors were implanted in the medial prefrontal cortex (mPFC; AP +1.95 mm from bregma, ML ±0.3 mm, DV -1.9 mm from dura) and the dorsal hippocampus (dHPC; AP -2.2 mm, ML ±1.8 mm, DV -2.0 mm). An LFP electrode was implanted in the CA1 pyramidal layer of the dorsal hippocampus (AP -1.8 mm from bregma, ML ±1.3 mm, DV -1.2 mm from dura), as close to the choline biosensor as possible (minimum distance, 500 μm) (Fig. S1).

Mice were anaesthetised with isoflurane (2%, delivered in oxygen at 1 L/min, Merial) and administered domitor (1 mg/kg, s.c.; Pfizer). Mice were positioned on the stereotaxic frame (Kopf) and general anaesthesia was maintained throughout the surgeries at 1% isoflurane in 1 L/min oxygen. Ophthalmic ointment (Alergan) was used to prevent their eyes from drying and reapplied as necessary. Under sterile conditions, craniotomies were drilled using a 0.7 mm steel burr (Fine Science Tools). Additional craniotomies were drilled to allow for four screws to be inserted into the skull and used as anchor points for the auxiliary electrode and the head cap. Under stereotaxic guidance, the choline biosensors, reference (0.235 mm diameter Teflon®-coated silver wire, Advent Research Materials) and LFP electrodes (0.2 mm diameter silver wire, Advent Research Materials) were inserted into place and secured with resin (Geristore Syringeable, DenMat). The auxiliary and LFP ground electrodes (8T silver wire, Advent Research Materials) were wrapped around skull screws (Fig. S1A). All electrodes were inserted into a six-pin plastic pedestal and secured using resin and dental cement (Meadway Rapid Repair, Mr. Dental Suppliers Ltd.) to make a robust head cap.

Post surgery, animals were administered an anti-sedative (antisedan, 5 mg/kg, s.c., Pfizer), anti-inflammatory (carprofen, 5 mg/kg, s.c., Pfizer), antibiotic (convenia, 5 mg/kg, s.c., Pfizer) and allowed to recover for 7 days.

***In vivo* constant potential amperometry and LFP recordings**

Each head-mounted 6-pin pedestal was tethered to a low noise, 4-channel potentiostat (EA164 QuadStat, eDAQ) and to a DP-301 differential amplifier (Warner Instruments) via a flexible six-core cable mounted through a swivel in the ceiling of the recording chamber to allow free movement of the animals throughout the recording cages.

Changes in extracellular tissue choline concentration were measured using constant potential amperometry (+700 mV). Day-matched homecage control recordings were performed at +200 mV, a potential value below the peak potential for H2O2 oxidation (~ +400 mV) and at which the contribution of the current generated by H2O2 oxidation at the sensor surface is minimized (Fig. S2D).

Hippocampal local field potentials were recorded using differential amplification. Low-pass (1 kHz) and high-pass (0.1 Hz) filters and an output gain of 1000 were used.

A 50 Hz low-pass digital filter was applied posthoc to eliminate mains AC noise from both the chemical and electrical signals. Choline and LFP data was digitised with a 16-channel eCorder unit (ED1621, eDAQ) and acquired with Chart (v5.5.18, eDAQ). All data was recorded at 1 kHz.

After application of the appropriate potential to the biosensors, the signal was allowed to settle for approximately 24 hours to ensure that the background current was completely stabilised. Once a baseline was obtained – stable current signal between 5 and 10 nA –, data was collected continuously for 12 hours during the light phase over a period of 5 consecutive days.

Randomized forced alternation T-maze test

Animals that recovered their pre-surgery weight were food restricted overnight and tested on an automated T-maze the following morning. All mice were tested at the same time of day over the five consecutive training days (Fig. 3A).

Entry of the mice into specific areas of the maze was detected using infrared beam breaks and passed to a microcontroller (Arduino Mega 2560). The Arduino software was instructed by MATLAB (The MathWorks,

Inc), which automatically controlled the maze protocol, allowing it to run without intervention from the experimenter.

Rewards were delivered by two pellet dispensers located at the end of each reward arm. To stop pellets from sliding far into the return arms, a small, soft barrier (2 mm high) was placed facing each pellet dispenser. Above the track there was an infrared video camera for the off-line tracking of animal location during maze performance and classification of trials.

Each trial on the maze comprised of two stages - a sample (forced) and a test (choice) phase. During sample trials the mice were released from the holding area at the base of the T-maze and allowed to run along the central arm. A sliding door at the choice point was positioned to guide the animal to turn and run towards one of the reward areas to receive a sucrose pellet. The animal then returned to the holding area and was held for a 5 second delay period (Ainge et al., 2007). During test trials, the door to the start arm (door 1) was opened and the animal was allowed a free choice between the two arms of the maze (both doors 2 and 3 open) (Fig. 3B). The average time, in seconds, taken for each mouse to travel between infrared beams on the central and choice arms during choice trials was defined as the average choice latency time.

The mice were rewarded for visiting the arm unexplored on the sample phase. At the end of the test phase, the animal was able to return to the start area, where it was confined for a 2 second delay period before the start of the next trial. Left/right allocations for the sample and choice runs were pseudo-randomised with no more than three consecutive sample runs to the same side.

Training on the task was not performed before the beginning of behavioral testing. During the active phase of this experiment animals were allowed to run 20 trials in a 60-minute period. No extra-maze cues or room configuration cues were used. At the end of each training session animals were returned to their chambers with *ad libitum* access to food and water.

Histology

At the end of the experiments, animals were deeply anesthetised with pentobarbital and perfused transcardially with 10% buffered paraformaldehyde (PFA). Animals were decapitated and the heads stored in PFA for 24 hours to ensure the brain tissue around the biosensors did not suffer excess damage upon removal of the head pedestal. Once the pedestal and attached sensors were carefully removed, brains were extracted and stored in PFA for histological processing.

To confirm biosensor electrode placement, serial 50 µm mPFC and dHPC sections were cut in the coronal plane using a cryostat.

To assess the possibility of gliosis around the biosensors, immunostainings against the ionised calcium-binding adapter molecule 1 (Iba-1) and glial fibrillary acidic protein (GFAP) were performed in tissue extracted one week post-implantation to match the starting point of recording and training of animals on the behavioural task (Fig S1C). 6 µm paraffin-embedded whole brain coronal sections were mounted on microscope slides. Sections were deparaffinised by a series of three 5-minute washes with xylene (Fisher Scientific), rehydrated in industrial methylated spirit (Fisher Scientific), and dried at 100°C for 20 minutes. Endogenous peroxidases were quenched for 10 minutes with 0.3% hydrogen peroxide (Sigma-Aldrich) in PBT (0.05% Tween 20 (ICN) in PBS (Sigma-Aldrich)). Non-specific background was blocked with goat serum (Vector Labs) for 30 minutes. Sections were then incubated in primary rabbit anti-GFAP (1:4000, AR020-5R Biogenex) or primary rabbit anti-Iba-1 (1:600, 019-19741 Wako) antibodies for 60 minutes at room temperature. The primary antibodies were rinsed and sections were incubated with secondary biotinylated goat anti-rabbit antibody (1:200, BA- 1000 Vector Labs) for 30 minutes at room temperature. All antibodies were diluted in PBT. Antibody labeling was achieved with ABC-horseradish peroxidase conjugate (Vector Labs) and 3,3'-diaminobenzidine chromagen (1:30, Vector Labs). Counterstaining was performed in haematoxylin (1:1, DAKO). Finally, sections were passed through industrial methylated spirit followed by xylene for dehydration. A coverslip was placed over each microscope slide using ClearVue mountant (Thermo Fisher Scientific).

All slides were imaged with an Aperio digital slice scanning system (Leica).

Locomotor activity analysis

Locomotor activity was monitored continuously using infrared cameras and analysed using a script from NIH Image (National Institutes of Health) as previously described (Richmond et al., 1998).

In these experiments, a difference of less than 50 pixels resulted in a ‘no-movement’ score and the mouse was judged to be sleeping. A difference of equal to or higher than 50 pixels resulted in a ‘movement’ score and the mouse was judged to be awake and moving.

Running speed during T-maze performance was assessed based on the time to travel between defined points on the maze at the start and end of each section. Running speed was considered to be zero in the holding area. To obtain the running speed over the duration of each maze training session, the average of the running speed over three consecutive trials was calculated at the beginning, middle and end of each session. Comparisons to choline current were made from recordings of the same epochs.

Sleep scoring

Arousal states were determined using the automated sleep scoring algorithm based on SCORETM (Van Gelder et al., 1991). These experiments were adapted to use a hippocampal LFP electrode instead of parietal and prefrontal skull screws, and locomotor activity in replacement of EMG. A mean of the integrated locomotor activity over the 10-second epochs was also used in arousal state assignment. Behavioral states were labelled according to the SCORETM protocol as wake, theta-dominated wake, NREM and REM. As EMG recordings could not be performed, quiet wakefulness was not included as a formal behavioral state for analysis. However, short periods of wakefulness with low locomotor activity (between 50 and 200 Δ pixels) that occurred between sleep cycles were labelled as quiet wakefulness. For analysis purposes, wake and theta-dominated wakefulness were combined and designated as Active Wake (AW). Epochs scored as REM or NREM sleep were corrected to Active Wake if the animals had moved during those epochs.

To plot changes in theta frequency power, raw LFP data were band-pass filtered between 0.7 and 30 Hz and downsampled to 100 Hz. Fourier power analysis was performed using the Chronux tool box. The ratio of the power in the theta (6–12 Hz) frequency band was calculated with a moving window (5 s, 0.5 s step) and z-normalised.

Data Analysis

Behavior-dependent event-triggered analysis

In vivo amperometry data were analysed using custom written MATLAB scripts. Data were low-pass filtered at 2 Hz and smoothed with a sliding window (window size = n+1, where n was the sampling frequency) to minimize noise levels. For each recording session in the homecage, three behavioral states were defined based on the scored data – Active Wake, REM and NREM. REM epochs were only included in the analysis if preceded by a minimum of 20 seconds (two consecutive 10 second bouts) of NREM. Each behavioral state was further split into a series of behavioral sequences to extract the patterns of behavioral-dependent acetylcholine release: long epochs of active wakefulness preceded by REM sleep ((REM-) **Active Wake**) or NREM sleep ((NREM-) **Active Wake**); REM sleep subsequently followed by wakefulness (**REM** (-Active Wake)); REM nested within NREM sleep (**REM** (-NREM)); and long NREM epochs subsequently followed by wakefulness (NREM (-Active Wake); Table S1). Occasionally, if NREM, a delta frequency oscillation-dominated state, was followed by a wake epoch with theta-dominated oscillations, transition bouts were incorrectly classified as REM. To avoid these false-positives to be classified as REM (-Wake), REM bouts were set to a minimum of 20 seconds in length – i.e. at least 2 consecutive bouts correctly scored as REM. Conversely, due to the very different LFP signatures of the two sleep states, short REM epochs flanked by NREM sleep could be accurately scored, allowing for the minimum REM bout length to be set to 10 seconds. The conditions for NREM epochs following nested REM bouts were stringent, requiring the minimum length of NREM to be 60 seconds. This allowed for a clear separation between REM and potentially mis-scored NREM–Theta-dominated wakefulness transition bouts.

For each behavioral state, an event window of at least 4 minutes was defined. Event-triggered analysis was performed by which all the timestamps marking the beginning of each epoch were aligned at the center of the 4-minute window and the corresponding choline signal extracted and stored in a multi-trial matrix. In the case of active wakefulness, a window of at least 12 minutes was set. To compensate for baseline differences between channels and individual transients, and to measure the change in current triggered by each behavioral state, data were baseline subtracted from the average value of the first 30-second period of the 4 minutes window, or from the first 60-120 seconds for analysis of active wake states. The choline current peak and the corresponding

timestamp of each event were extracted. The difference between scored behavior onset and the time of peak for the mPFC and dHPC was calculated to determine the time difference between events in each brain region. For plotting purposes, all traces were smoothed using a 35-point moving window.

Table S1. List of behavioral sequences defined for behavior-dependent event-triggered analysis*. Related to Figure 2.

Behavioral State	Min. epoch length (s)	Preceding State	Min. epoch length (s)	Following State	Min. epoch length (s)
(REM-) **Active Wake**	600	REM	20	n/a	n/a
(NREM-) **Active Wake**	600	NREM	20	n/a	n/a
**REM** (-Wake)	20	NREM	20	Wake	10
**REM** (-NREM)	10		20	NREM	60
**NREM** (-Wake)	60	n/a	n/a	Wake	10

* main behavioral state in bold

To measure changes in current during training on the T-maze task, data were smoothed with a 300-point moving window to remove noise artifacts and the maximum current was extracted for each training day. To calculate the change relative to baseline, data were normalized by subtracting the average current value of the 60 seconds recorded prior to placing each animal on the maze. Data were normalized by the standard deviation of choline currents within each animal to enable statistical comparisons across animals.

Phasic transients analysis

To eliminate the slow change in the choline signal observed during maze testing, the data were detrended in MATLAB by removing a continuous, piecewise linear trend from the data vector between linearity breakpoints, thereby flattening the traces without affecting the high frequency component of the data containing the fast transients. Detection of phasic choline transients was done using the ClampFit template-matching tool (Molecular Devices Corporation) (Clements and Bekkers, 1997). Template waveforms were created for each implanted biosensor by averaging 3 – 6 large events detected by visual inspection. Events that were part of equal and opposite positive and negative going deflections were deemed non-biological and excluded. Events smaller than 3 times the average standard deviation of the raw data (3σ) for each animal were also excluded post hoc. Average standard deviations were similar during T-maze training or in the homecage with the sensor potential set at +700 mV or +200 mV and were consistent between mice (0.06 ± 0.004 nA for mPFC (n = 30) and 0.06 ± 0.004 nA for dHPC (n = 32) for 6 mice, all pairwise comparisons n.s., ANOVA with Tukey HSD posthoc correction). A comparison of the amplitude frequency distribution of events detected at biosensor potentials of +200 mV and +700 mV both in the homecage and on the maze revealed that a 3σ amplitude threshold excluded almost all template matched events occurring at +200mV and therefore not choline-mediated (Fig. 4B).

Fast transients larger than the threshold were assigned to maze sections according to the position of the mouse on the maze when each transient occurred. To calculate the proportion of transients that occurred in each maze section, the data were normalised to the total number of transients detected in each brain region. This was plotted as a color plot smoothed with a 2D Gaussian low-pass filter. The number of events detected in each maze section was compared against the number of events detected in all other coordinates. To calculate the proportion of coordinated events that occurred in each maze section, the data were normalized to the total number of coordinated transients detected. Transients detected in the reward, choice, middle arm or holding area were further split into the categories of forced, correct-choice, and wrong-choice trials based on which phase of the maze and choice outcome the transients coincided with. Data from each animal were normalised to the total number of trials of each type before averaging across animals. The proportion of coordinated events that occurred during forced, correct-choice, and wrong-choice trials was calculated by normalizing the number of coordinated events detected in each trial type to the total number of trials of each type.

Statistical analysis

Statistical significance and normality tests were performed using tests in SPSS (v23.0.0.2, IBM). Where data did not pass the Levene's test for equal variance between groups, one-way Welch's ANOVA was used for all multiple comparisons tests with Games-Howell posthoc adjustment. Otherwise, a standard one-way ANOVA was used with Tukey HSD or Dunnett posthoc adjustment or two-tailed paired t-test for within animal comparison of +700mV to +200mV REM transients. Two-tailed Mann–Whitney tests were used for comparisons between two independent groups. Unless otherwise stated, data are reported as means ± standard error of the mean (SEM). ns denotes $p>0.05$, * denotes $p<0.05$, ** denotes $p<0.01$, *** denotes $p<0.001$.
